# Sulforaphane downregulated fatty acid synthase and inhibited microtubule-mediated mitophagy leading to apoptosis

**DOI:** 10.1038/s41419-021-04198-2

**Published:** 2021-10-07

**Authors:** Yuting Yan, Yan Zhou, Juntao Li, Zhongnan Zheng, Yabin Hu, Lei Li, Wei Wu

**Affiliations:** 1 Department of Biochemistry and Molecular Biology, School of Basic Medical Sciences, Capital Medical University, Beijing, China; 2 Beijing Key Laboratory for Tumor Invasion and Metastasis, Beijing, China; 3 State Key Laboratory of Cardiovascular Disease, Fuwai Hospital, Beijing, 100037 China; 4 Central Laboratory, Capital Medical University, Beijing, China; 5 Capital Medical University, No. 10, Xitoutiao, Beijing, 100069 China

**Keywords:** Fatty acids, Non-small-cell lung cancer, Mitophagy

## Abstract

We previously demonstrated that sulforaphane (SFN) inhibited autophagy leading to apoptosis in human non-small cell lung cancer (NSCLC) cells, but the underlying subcellular mechanisms were unknown. Hereby, high-performance liquid chromatography-tandem mass spectrometry uncovered that SFN regulated the production of lipoproteins, and microtubule- and autophagy-associated proteins. Further, highly expressed fatty acid synthase (FASN) contributed to cancer malignancy and poor prognosis. Results showed that SFN depolymerized microtubules, downregulated FASN, and decreased its binding to α-tubulin; SFN downregulated FASN, acetyl CoA carboxylase (ACACA), and ATP citrate lyase (ACLY) via activating proteasomes and downregulating transcriptional factor SREBP1; SFN inhibited the interactions among α-tubulin and FASN, ACACA, and ACLY; SFN decreased the amount of intracellular fatty acid (FA) and mitochondrial phospholipids; and knockdown of FASN decreased mitochondrial membrane potential (ΔΨm) and increased reactive oxygen species, mitochondrial abnormality, and apoptosis. Further, SFN downregulated mitophagy-associated proteins Bnip3 and NIX, and upregulated mitochondrial LC3 II/I. Transmission electron microscopy showed mitochondrial abnormality and accumulation of mitophagosomes in response to SFN. Combined with mitophagy inducer CCCP or autophagosome–lysosome fusion inhibitor Bafilomycin A1, we found that SFN inhibited mitophagosome–lysosome fusion leading to mitophagosome accumulation. SFN reduced the interaction between NIX and LC3 II/I, and reversed CCCP-caused FA increase. Furthermore, knockdown of α-tubulin downregulated NIX and BNIP3 production, and upregulated LC3 II/I. Besides, SFN reduced the interaction and colocalization between α-tubulin and NIX. Thus, SFN might cause apoptosis via inhibiting microtubule-mediated mitophagy. These results might give us a new insight into the mechanisms of SFN-caused apoptosis in the subcellular level.

## Introduction

We reported that sulforaphane (SFN) metabolite-induced microtubule disruption contributed to the inhibition of cell proliferation and autophagy leading to apoptosis [1, 2]. Meanwhile, SFN might cause swollen mitochondria in non-small cell lung cancer (NSCLC) [3]. In a separate study, we found that SFN might inhibit mitophagy and mitochondrial glucose metabolism via impeding the trafficking and translocation of damaged proteins into mitochondria (unpublished data). Hence, some deeper mechanisms involved in SFN-triggered mitochondrial dysfunction and metabolic abnormality might result in apoptosis. Besides, report showed that abnormal lipid metabolism might cause cell apoptosis [4]. De novo synthesis is the main pathway for cancer cells to produce fatty acids (FAs); FA synthase (FASN) is highly expressed in various cancers including lung cancer and low production of FASN in NSCLC patients have longer survival [5–7]. Study showed that downregulation of FASN production caused apoptosis in tumor cells rather than normal cells [8]. FAs are essential components of biofilm lipids and are important substrates for energy metabolism [9–11]; tumor cells require large amounts of FAs for rapid division and proliferation [12, 13].

Glucose uptake by tumor cells produces pyruvate by glycolysis, a small part of pyruvate molecules enter the mitochondria to undergo oxidative decarboxylation to form citric acids. Citric acids were transported out of mitochondria and catalyzed by ATP citrate lyase (ACLY) to release acetyl CoA. Acetyl CoA carboxylase (ACACA) catalyzes acetyl CoA to form malonyl CoA. In the presence of nicotinamide adenine dinucleotide phosphate, acetyl CoA interacts with malonyl CoA to synthesize FAs by FASN [14]. FA metabolites might act as signal molecules involved in tumor growth or participate in protein modification after translation [8]. Therefore, the interference of FA synthesis by inhibiting FA synthesis-related molecules might help us establish an effective anti-cancer therapy.

Studies showed that cruciferous plant-derived SFN inhibited lipid production and induced apoptosis in prostate cancer cells [13, 15]. SFN induced apoptosis of NSCLC cells in a concentration-dependent manner at 20 μM concentration [3]. Just recently, we reported that the combination of SFN metabolites and paclitaxel reduced the dosage and restored sensitivity of paclitaxel-resistant cells to paclitaxel [16]. We also found that SFN metabolites induced apoptosis by inhibiting microtubule-mediated autophagy in NSCLC [1]. Microtubules are the dimers formed by α-tubulin, β-tubulin, and a small part of microtubule-binding proteins, such as microtubule-associated protein 1 light chain 3 (LC3 II/I) and stathmin-1 [17]. However, it was not clear whether SFN-caused microtubule disruption contributed to inhibition of FA synthesis and mitophagy. In particular, whether the inhibition of mitophagy regulated FA production leading to apoptosis was unknown.

Bafilomycin A1 (Baf-A1) is an autophagy inhibitor that blocks the fusion of autophagosome to lysosome. We previously reported that, combined with Baf-A1, SFN metabolites inhibited the fusion of autophagosome to lysosome and caused the accumulated autophagy flux LC3 II/I leading to apoptosis [1]. LC3 II/I located on the autophagic membrane and were autophagy marker proteins [18]. Mitophagy helps cells to remove damaged mitochondria and maintain cellular proteostasis [19, 20]. Mitophagy is mainly mediated by the PINK/Parkin, BNIP3/NIX, and FUNDC1 signaling pathways. NIX is a NIP3 protein on mitochondria in the Bcl-2 family. BNIP3 is a member of the Bcl-2 protein family and belongs to the BH3 subfamily containing only the BH3 domain [21, 22]. The damaged mitochondria are surrounded by a bilayer membrane to form mitophagosomes. NIX on the outer membrane of mitochondria recruits LC3 II/I to form autophagosomes enveloping damaged mitochondria; the phagocytosis of damaged mitochondria maintains the homeostasis of cells [23, 24]. Consequently, SFN might inhibit the fusion of mitophagosomes to lysosomes and the recruitment of LC3 II/I to NIX [25].

Taken together, we hypothesized that SFN inhibited microtubule-mediated FA synthesis disrupting mitochondrial membrane structures and inhibited microtubule-mediated mitophagy leading to apoptosis. These studies might help us understand the deeper mechanisms that SFN produced in the subcellular levels and establish efficient anti-cancer therapies.

## Results

### SFN-caused apoptosis might result from the downregulation of FA synthesis-associated proteins and microtubule-associated proteins

**Fig. 1:**
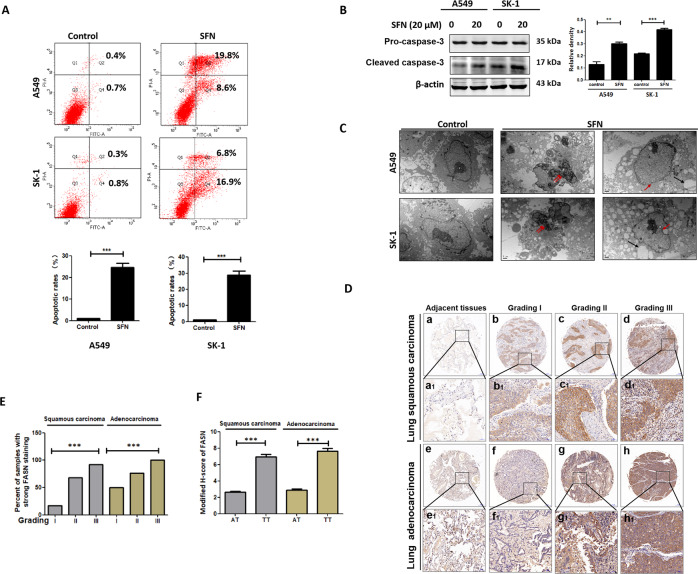
SFN-induced apoptosis might result from the inhibition of microtubule-mediated FA synthesis. **A** Both of A549 and SK-1 cells were treated with SFN (20 µM) for 24 h, then the cells were collected and the percentages of cell apoptosis were analyzed by a flow cytometer. **B** Caspase-3 proteins were analyzed via western blotting in A549 and SK-1 cells treated with SFN (20 µM). **C** After induction with 20 μM SFN, both A549 and SK-1 cells were collected and fixed. Cell pellets were cut into thin slices and stained with uranium acetate and lead nitrate. Cell morphology was viewed with TEM. Double red arrows marked nuclear fragments, black arrows indicate sporadic vacuoles, and red arrows indicate membrane disruption. Scale bar: 1 μm. **D** Levels of FASN were assessed in the microarray tissues of lung adenocarcinoma and lung squamous carcinoma. FASN levels in the 150 adjacent tissues were tested as the controls (**A**, **E**) and 150 cancer tissues with three malignant grades (**B**–**D**, **F**–**H**) were used. Upper panel: magnification ×50, scale bars: 500 μm. Lower panel: magnification ×200, scale bars: 100 μm. **E** Percentages of samples with strong FASN staining in 75 tumor tissues, which were divided into three malignant grades. **F** The comparison of H scores for FASN accumulation between adjacent and tumor tissues were done in lung adenocarcinoma and lung squamous carcinoma (AT: 75 adjacent tissues; TT: 75 tumor tissues). Data were shown as means ± SEM (*n* ≥ 3). **P ≤ 0.001; ***P ≤ 0.0001.

These results by high-performance liquid chromatography-tandem mass spectrometry (HPLC-MS/MS) analysis showed that SFN affected the production of lipoproteins, membrane proteins, and autophagy-, apoptosis- and microtubule-associated proteins (Table 1). Further, the rates of apoptosis were increased and cleaved caspase-3 level was increased after 24 h treatment with SFN (Fig. 1A, B). Cell apoptotic features, such as chromatin condensation, apoptotic bodies, and vacuolization were viewed by a transmission electron microscope (TEM) (Fig. 1C). Tissue microarray analysis showed a positive correlation of FASN level to the malignancy of NSCLC (Fig. 1D). FASN level is positively correlated to the malignant grades of tumor cells in both lung squamous cell carcinoma and lung adenocarcinoma (Fig. 1E). The correlation of FASN expression to pathological grading of NSCLC was analyzed by calculating the H-score (Table 2). The level of FASN in tumor tissues is higher than that in adjacent tissues (Fig. 1F). These results suggested that SFN-caused apoptosis might result from the downregulation of FA synthesis-associated proteins and the dysfunction of microtubule- and autophagy-associated proteins, and membrane proteins.

**Table 1:** HPLC-MS/MS analysis showed that SFN affected the production of lipoproteins, membrane proteins, and autophagy-, apoptosis-, and microtubule-associated proteins.

Lipid metabolism	Membrane	Microtubule	Apoptosis	Autophagy
Downregulated proteins				
Fatty acid synthase	Isoform 3 of calcium-binding mitochondrial carrier protein	Tubulin β-4B chain	Pleckstrin homology-like domain family A member 3	Phosphatidylinositol 3-kinase catalytic subunit type 3
Isoform 3 of 1-phosphatidylinositol 4,5-bisphosphate phosphodiesterase β-4	Oxysterol-binding protein-related protein 3	Tubulin α-4A chain	Protein JTB	Phosphatidylinositol 4,5-bisphosphate 3-kinase catalytic subunit β-isoform
Isoform 3 of cytosolic phospholipase A2 epsilon	Ras-related protein	Tubulin α-1C chain	Tensin-4	Ubiquitin-like modifier-activating enzyme ATG7
Acyl-protein thioesterase 2	Peroxisomal membrane protein	Isoform 3 of echinoderm microtubule-associated protein-like 2	Isoform 2 of insulin-like growth factor-binding protein 3	RB1-inducible coiled-coil protein 1
15-Hydroxyprostaglandin dehydrogenase [NAD+]	Phosphatidylinositol glycan anchor biosynthesis class U protein	β5-Tubulin		Isoform 5 of autophagy-related protein 13
Upregulated proteins				
Apolipoprotein A-I	Isoform 2 of ATP-binding cassette subfamily B member 7	Dynein heavy chain 2	BAG family molecular chaperone regulator 3	Ubiquitin-like protein ATG12
Apolipoprotein C-III	Essential MCU regulator	Kinesin-like protein KIF23	Cytoskeleton-associated protein 2	Isoform 2 of autophagy-related protein 2 homolog A
Serine incorporator 1	ATP synthase subunit e	Isoform 2 of targeting protein for Xklp2	Tumor necrosis factor receptor superfamily member 10D	
	MICOS complex subunit	Nucleolar and spindle-associated protein 1	Cytochrome *c*	
			Programmed cell death protein 7

**Table 2:** Correlation of FASN expression to clinicopathological characteristics of lung cancer patients.

Variable	Lung squamous carcinoma				Variable	Lung adenocarcinoma			
	All patients	Low	High	*P*-value		All patients	Low	High	*P*-value
Gender				0.458	Gender				0.567
Male	71	19	52		Male	39	10	29	
Female	4	2	2		Female	35	7	28	
Age (years)				0.893	Age (years)				0.136
>60	40	12	28		>60	41	12	29	
≤60	35	10	25		≤60	34	5	29	
Differentiation				0.001*	Differentiation				0.003*
I	6	5	1		I	14	7	7	
II	44	14	30		II	42	10	32	
III	25	2	23		III	19	0	19	
Staging				0.144	Staging				0.838
IA	9	1	8		IA	14	4	10	
IB	14	4	10		IB	23	5	18	
IIA	13	7	6		IIA	14	2	12	
IIB	10	3	7		IIB	4	1	3	
IIIA	22	6	16		IIIA	11	4	7	
IIIB	2	0	5		IIIB	3	0	3	
IV	5	0	5		III	2	0	2	
					IV	4	1	3	

Low (Score 0–4), High (Score 5–12).

*p < 0.05 was defined as statistically significant.

### SFN inhibited the polymerization of microtubules

**Fig. 2:**
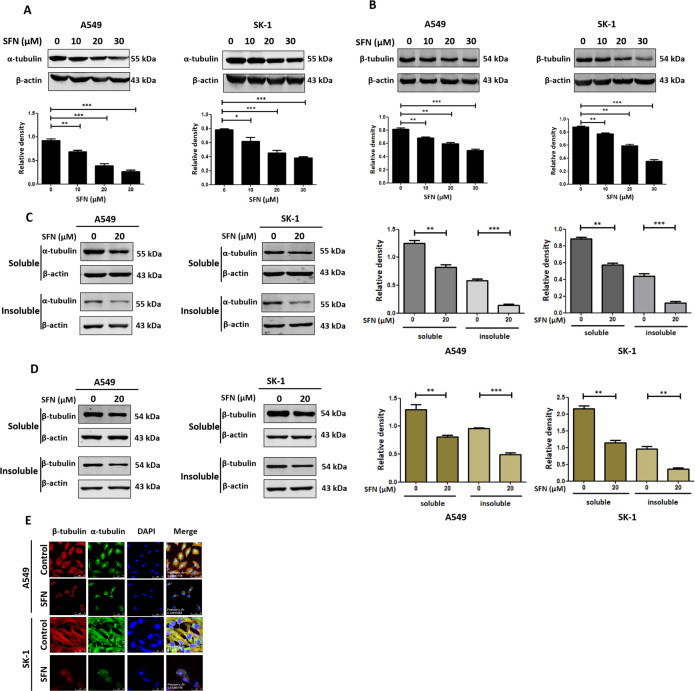
SFN caused microtubule disruption by breaking α-tubulin and β-tubulin dimer. **A**, **B** The accumulation of α-tubulin and β-tubulin was detected by western blotting after the treatment of 0, 10, 20, and 30 μM SFN for 24 h in A549 and SK-1 cells. **C**, **D** Both A549 and SK-1 cells were treated with/without 20 μM SFN for 24 h. Then the accumulation of soluble and insoluble α-tubulin (**C**) or β-tubulin (**D**) was determined by western blotting in both A549 and SK-1 cells. **E** Immunofluorescence and confocal microscopy were employed to view the colocalization of α-tubulin to β-tubulin. Scale bar: A549: 75 µm, SK-1: 25 µm. Data were shown as means ± SEM (*n* ≥ 3). **P ≤ 0.001; ***P ≤ 0.0001.

Cells were treated with different concentrations of SFN for 24 h. Results showed that SFN significantly downregulated α-tubulin in a concentration-dependent manner (Fig. 2A). Meanwhile, β-tubulin was also downregulated in a concentration-dependent manner (Fig. 2B). Microtubule polymerization assays showed that the amounts of both soluble and insoluble α-tubulin or β-tubulin were lower vs. control, indicating that the SFN depolymerized the microtubules (Fig. 2C, D). Immunofluorescence staining showed that SFN disrupted microtubules and reduced the colocalization of α-tubulin to β-tubulin (Fig. 2E). In a separate study, we found that SFN reduced the interaction between α-tubulin and β-tubulin (unpublished data). These results indicated that SFN inhibited microtubule polymerization leading to microtubule disruption.

### SFN inhibited microtubule-mediated FA synthesis via activating 26S proteasome

**Fig. 3:**
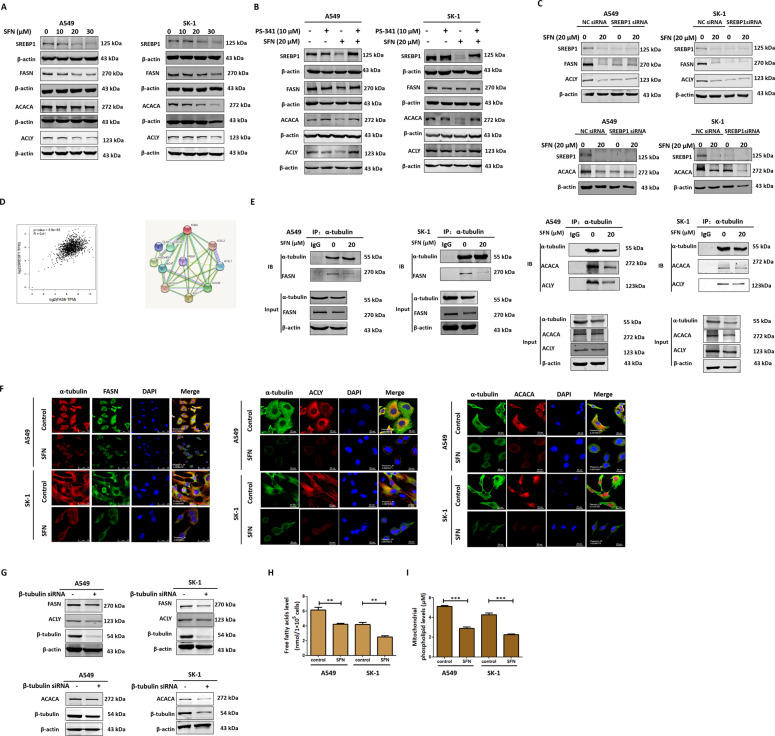
SFN inhibited microtubule-mediated FA synthesis via activating 26S proteasome. **A** Both A549 and SK-1 cells were treated with 0, 10, 20, and 30 μM SFN for 24 h. Western blotting was used to analyze the accumulation of SREBP1, FASN, ACACA, and ACLY. **B** Both A549 and SK-1 cells were pretreated with PS-341 (10 μM) for 2 h, then treated with SFN (20 μM) for 24 h. The accumulation of SREBP1, FASN, ACACA, and ACLY was analyzed by western blotting. **C** After knockdown of SREBP1, western blotting was used to analyze ACACA, FASN, and ACLY accumulation. **D** GEPIA database was used to analyze the correlation between transcription factor SREBP1 and FASN. If R closer is to “1,” the correlation between the two proteins is higher. Via the String net, we analyzed the interactions among FASN and related partners. **E** After induction with 20 µM SFN for 24 h, coimmunoprecipitation was employed to detect the interaction between α-tubulin and FASN, ACACA, and ACLY. **F** Immunofluorescence and confocal microscopy were employed to observe the colocalization of α-tubulin to FASN (scale bar: A549: 75 µm, SK-1: 25 µm), ACACA (scale bar: 25 µm), ACLY (scale bar: 25 µm). **G** After knockdown of β-tubulin, western blotting was used to analyze ACACA, FASN, and ACLY accumulation. **H** Determination of total free FA content after induction with 20 µM SFN for 24 h. **I** After induction with 20 µM SFN for 24 h, mitochondrial phospholipid content was detected by kit. Data were shown as means ± SEM (*n* ≥ 3). *P ≤ 0.01, **P ≤ 0.001, ***P ≤ 0.0001.

After treatment with gradient concentrations of SFN for 24 h, the results showed that SREBP1, FASN, ACACA, and ACLY were downregulated in a concentration-dependent manner (Fig. 3A). Combined with proteasome inhibitor PS-341, the results show that SFN degraded SREBP1, FASN, ACACA, and ACLY via activating 26S proteasome (Fig. 3B). After knockdown of SREBP1 production, the FA-related proteins ACACA, FASN, and ACLY were downregulated (Fig. 3C). These results showed that SFN degraded the key enzymes in FA synthesis through two pathways, the 26S-proteasomal pathway and the downregulation of SREBP1, as a transcriptional factor for FASN production. Correlation analysis showed that level of SREBP1 had a correlation to FASN level and the level of either ACACA or ACLY, or FASN was closely correlated to FA synthesis (Fig. 3D). Moreover, we found that SFN inhibited the interaction between α-tubulin and FASN, ACACA, and ACLY (Fig. 3E); meanwhile, SFN lowered the colocalization of FASN, ACACA, and ACLY to α-tubulin (Fig. 3F). After knockdown of β-tubulin production, the FA-related proteins ACACA, FASN, and ACLY was downregulated (Fig. 3G). These results showed that disrupted microtubule led to decrease of expression level of FA-related proteins. The levels of FAs and mitochondrial phospholipids were significantly reduced in response to SFN (Fig. 3H, I). Therefore, SFN might lower the production of mitochondrial phospholipids by inhibiting microtubule-mediated FA synthesis leading to the dysfunction of mitochondrial membranes.

### SFN-triggered downregulation of FASN level contributed to apoptosis

**Fig. 4:**
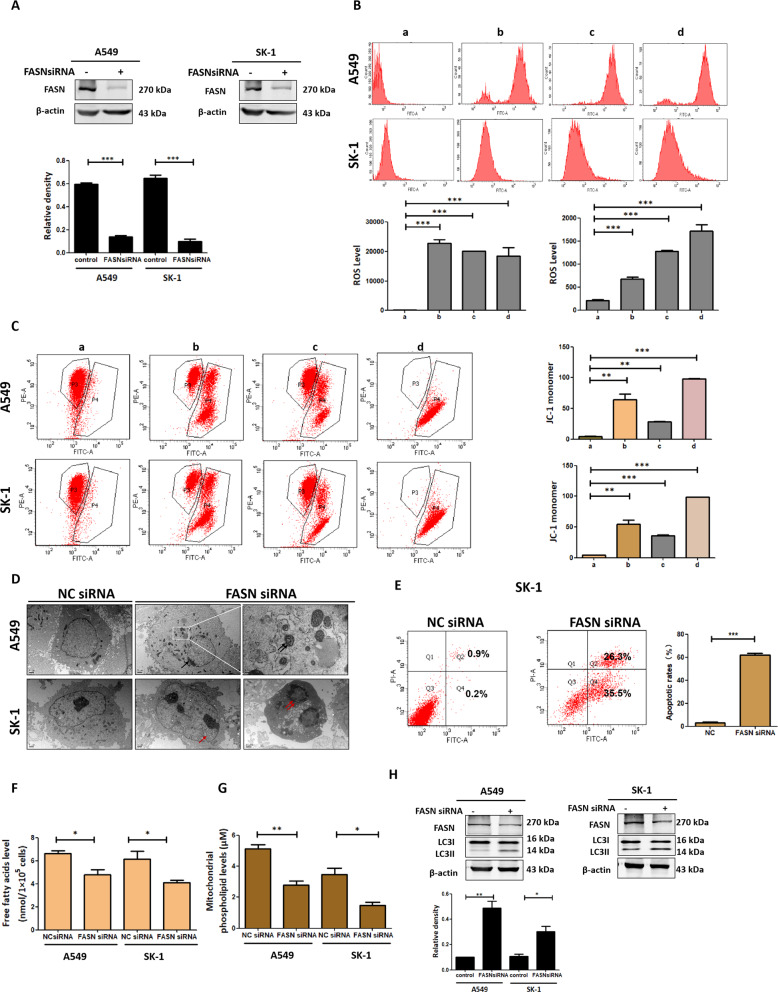
SFN-triggered downregulation of FASN level contributed to apoptosis. **A** FASN accumulation was detected by western blotting after knockdown of FASN via siRNA, both in A549 and SK-1 cells. **B** Flow cytometry was used to detect ROS levels after knocking down FASN mRNA. (a: NC siRNA, b: FASN siRNA, c: 20 μM SFN, d: FASN siRNA plus 20 μM SFN). **C** Flow cytometry was used to detect JC-1 levels after knocking down FASN mRNA in cells (a: NC siRNA, b: FASN siRNA, c: 20 μM SFN, d: FASN siRNA plus 20 μM SFN). **D** After knocking down FASN mRNA in cells, cells were observed via TEM. Red arrows indicate membrane disruption, black arrows indicate chromatin edge aggregation, double black arrows indicate swollen mitochondria and double red arrows indicated nuclear fragments. Scale bar: 1 μm. **E** Cells were transfected with NC siRNA or FASN siRNA, flow cytometry was used to detect apoptosis rates. **F** Determination of total free FA content after FASN was knocked down. **G** Mitochondrial phospholipid content was detected by kit after FASN was knocked down. **H** After knockdown of FASN level, western blotting was used to analyze LC3 II/I accumulation. Data were shown as means ± SEM (*n* ≥ 3). *P ≤ 0.01; **P ≤ 0.001; ***P ≤ 0.0001.

The FASN level was successfully knocked down via small interfering RNA (siRNA) interference leading to the elevated intracellular reactive oxygen species (ROS) level and downregulation of mitochondrial membrane potential (ΔΨm) (Fig. 4A–C). Abnormal cell membrane structures, swollen mitochondria, and apoptotic features were viewed (Fig. 4D). Knockdown of FASN production induced cell apoptosis (Fig. 4E). The levels of FA and mitochondrial phospholipid were significantly reduced in the FASN siRNA group vs. control (Fig. 4F, G). After knockdown of FASN production, the microtubule-associated protein LC3 II/I was upregulated (Fig. 4H). These results indicated that inhibition of FASN production might reduce cellular FA content, thereby reducing intracellular phospholipid level, leading to mitochondrial membrane instability and apoptosis.

### SFN inhibited microtubule-mediated mitophagy and the fusion of mitophagosomes to lysosomes

**Fig. 5:**
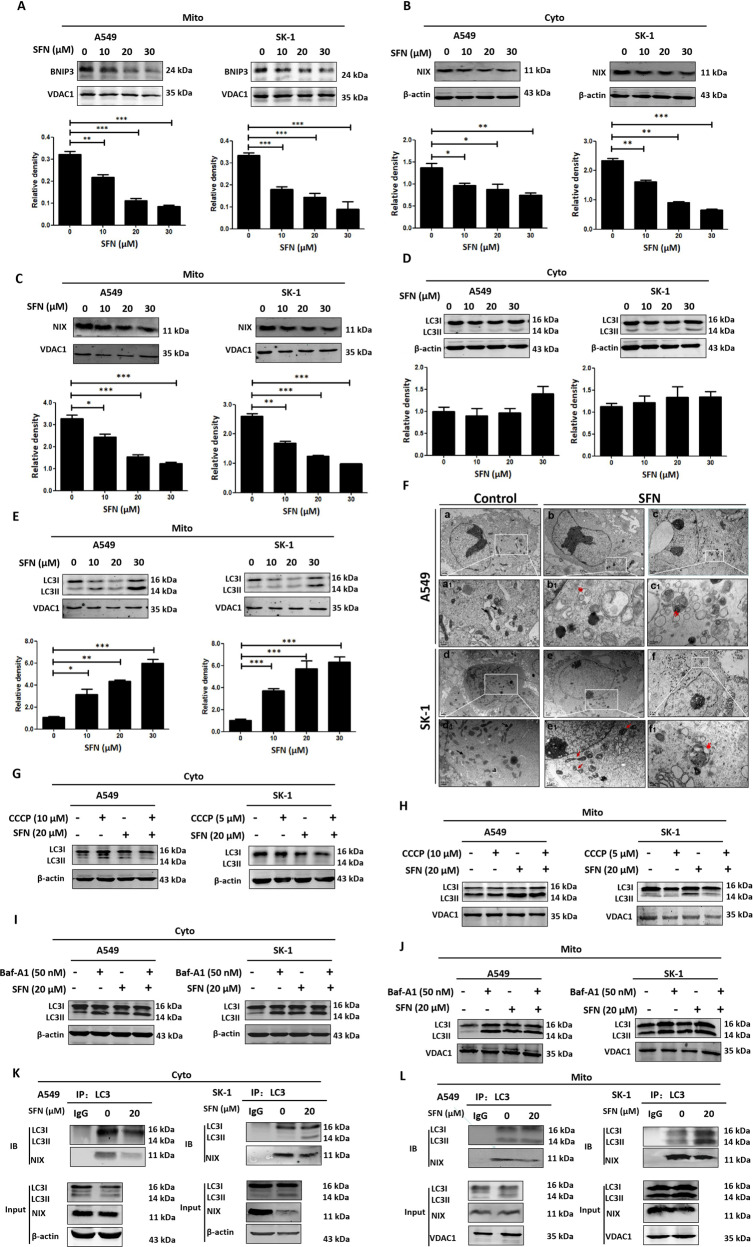
SFN inhibited microtubule-mediated mitophagy and the fusion of mitophagosomes to lysosomes. After SFN was used at a designated concentration for 24 h, the mitochondrial isolation kit was used to separate the cytoplasm and mitochondrial protein lysates. Western blotting was used to analyze BNIP3 level in the mitochondria. **B**, **C** After SFN was used at a designated concentration for 24 h, the mitochondrial isolation kit was used to separate the cytoplasmic and mitochondrial proteins. Western blotting was used to analyze NIX level in the cytoplasm (**B**) and mitochondria (**C**). **D**, **E** After SFN was used at a designated concentration for 24 h, the mitochondrial isolation kit was used to separate the cytoplasmic and mitochondrial proteins. Western blotting was used to analyze LC3 II/I accumulation in the cytoplasm (**D**) and mitochondria (**E**). **F** Cells were treated with/without 20 µM SFN for 24 h. The images via TEM showed the mitophagosomes; double red arrows marked mitophagosomes; black arrows marked normal mitochondria; red arrows marked swollen mitochondria. Scale bar: 1 μm (a–f), 0.5 μm (a_1_–f_1_). **G**, **H** Both A549 and SK-1 cells were pretreated with CCCP (10 or 5 μM) for 2 h, then treated with SFN (20 μM) for 24 h, the mitochondrial isolation kit was used to separate the cytoplasmic and mitochondrial proteins. The cytoplasm (**G**) or mitochondria (**H**) accumulation of LC3 II/I was analyzed by western blotting. **I**, **J** Both A549 and SK-1 cells were pretreated with Baf-A1 (50 nM) for 2 h, then treated with SFN (20 μM) for 24 h, the mitochondrial isolation kit was used to separate the cytoplasm and mitochondrial proteins. The cytoplasm (**I**) or mitochondria (**J**) accumulation of LC3 II/I was analyzed by western blotting. **K**, **L** After SFN was used at a designated concentration for 24 h, the mitochondrial isolation kit was used to separate the cytoplasmic and mitochondrial proteins. The binding of LC3 II/I to NIX was detected in both cytoplasm (**K**) and mitochondria (**L**) by coimmunoprecipitation. Data were shown as means ± SEM (*n* ≥ 3). *P ≤ 0.01; **P ≤ 0.001; ***P ≤ 0.0001.

We found that mitophagy-associated protein BNIP3 localized to mitochondria and SFN downregulated BNIP3 level (Fig. 5A), and the mitochondrial mitophagy-associated protein NIX was expressed in both the mitochondria and cytoplasm, and the expressions were inhibited by SFN in a concentration gradient (Fig. 5B, C). The accumulated microtubule-associated protein LC3 II by SFN was mainly present in mitochondria and SFN treatment had no significant effect on LC3 II/I in cytoplasm (Fig. 5D, E). Mitophagosomes in the cells were viewed after treated with SFN, whereas autolysosome was rarely visible (Fig. 5F). After co-treated with the mitophagy inducer carbonylcyanide 3-chlorophenylhydrazone (CCCP), SFN reversed LC3 II/I downregulation (Fig. 5G, H). After co-treated with Baf-A1, SFN did not make a significant change in LC3 II/I level (Fig. 5I, J). These results indicated that SFN inhibited mitophagy by hindering the fusion of mitophagosomes to lysosomes. Coimmunoprecipitation showed that SFN reduced the interaction between NIX and LC3 II/I in the cytoplasm and mitochondria, and inhibited the initiation of mitophagy (Fig. 5K, L). These results showed that SFN inhibited mitophagy by lowering the expression of mitophagy-associated proteins and the interaction between microtubule-associated protein LC3 II/I and mitophagy-associated proteins inhibiting the degradation of mitophagosomes.

### SFN induced apoptosis by inhibiting microtubule-mediated FA synthesis and mitophagy

**Fig. 6:**
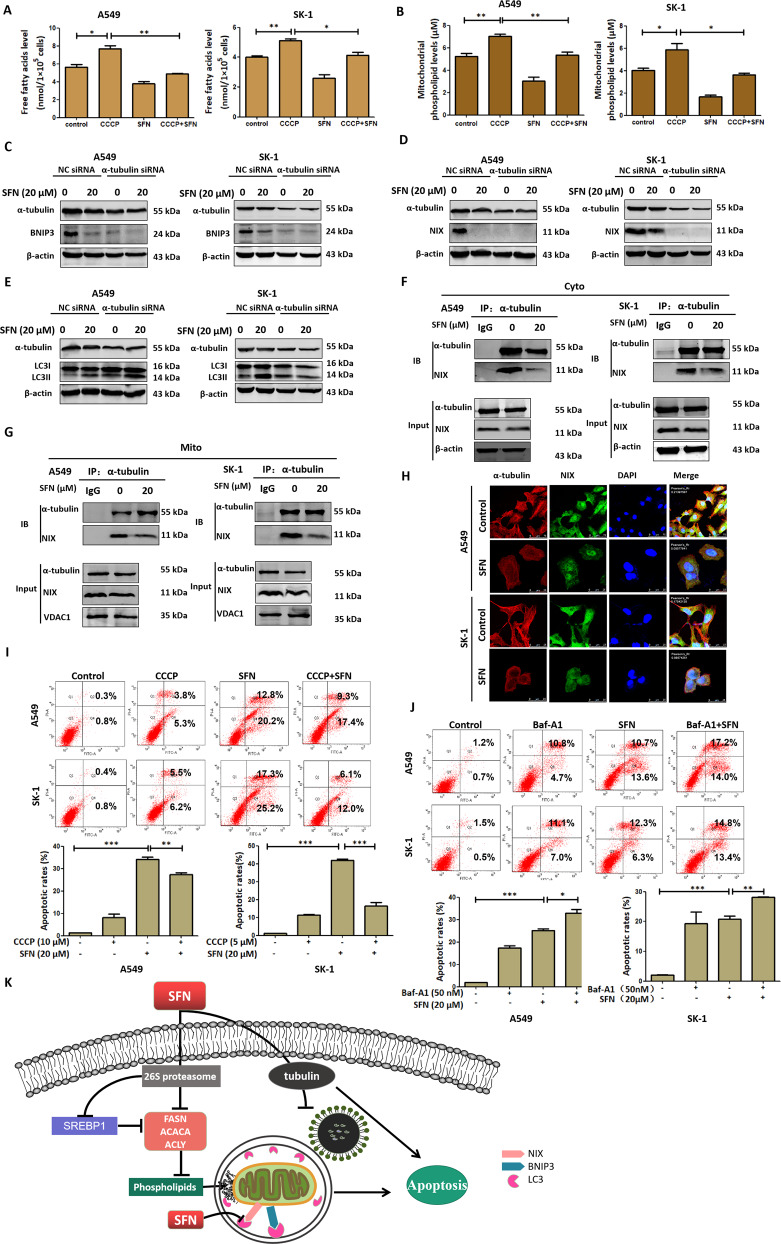
SFN induced apoptosis by inhibiting microtubule-mediated FA synthesis and mitophagy. **A**, **B** Both A549 and SK-1 cells were pretreated with CCCP (10 or 5 μM) for 2 h, then treated with SFN (20 μM) for 24 h, determination of total free FA (**A**) and mitochondrial phospholipid content (**B**) was determined. **C**–**E** Both A549 and SK-1 cells were transfected with NC siRNA or α-tubulin siRNA, and treated with SFN (20 μM) for 24 h, the accumulation of BNIP3 (**C**) or NIX (**D**), or LC3 I /II (**E**) was determined by western blotting. **F**, **G** Both A549 and SK-1 cells were treated with SFN (20 μM) for 24 h, the mitochondrial isolation kit was used to separate the cytoplasmic and mitochondrial proteins. Then binding of α-tubulin to NIX was detected both in the cytoplasm (**F**) and mitochondria (**G**) by coimmunoprecipitation. **H** Immunofluorescence and confocal microscopy were employed to observe the colocalization of α-tubulin to NIX. Scale bar: A549: 75 µm, SK-1: control, 25 µm, SK-1: SFN, 25 µm. **I**, **J** Both A549 and SK-1 cells were pretreated with CCCP (10 or 5 μM) (**I**) or Baf-A1 (50 nM) (**J**) for 2 h, then treated with SFN (20 μM) for 24 h, then the cells were collected and the percentage of apoptosis rates were analyzed by flow cytometer via Annexin V-FITC/PI Apoptosis Detection Kit. **K** A possible schematic of the involved signal pathways that SFN induced apoptosis via inhibiting microtubule-mediated FA synthesis and mitophagy in NSCLC cells. Data were shown as means ± SEM (*n* ≥ 3). *P ≤ 0.01; **P ≤ 0.001; ***P ≤ 0.0001.

After the cells were treated with the mitophagy inducer CCCP, FA levels were increased. Combined with SFN, the increased FA levels were reversed (Fig. 6A). The analysis via Phospholipid Kits showed the consistent results (Fig. 6B). These indicated that SFN-inhibited mitophagy contributed the decrease of FA. Reversely, the decrease might inhibit mitophagy. After knocking down of α-tubulin, mitophagy markers BNIP3 and NIX levels were downregulated (Fig. 6C, D). Meanwhile, LC3 II/I levels were upregulated (Fig. 6E). These indicated that SFN inhibited the binding of mitophagosome to lysosome by decreasing α-tubulin. Coimmunoprecipitation showed that the interaction between α-tubulin and NIX was downregulated in both the mitochondria and cytoplasm (Fig. 6F, G). Confocal microscopy observation showed that SFN inhibited the colocalization of α-tubulin to NIX (Fig. 6H). These results indicated that SFN inhibited mitophagy regulating FA level via a microtubule-mediated way. Flow cytometry showed that cell apoptosis rates were decreased after co-treated with CCCP and the apoptosis rates were increased after co-treated with Baf-A1 (Fig. 6I, J). Taken together, these data demonstrated that SFN inhibited microtubule-mediated FA synthesis and mitophagy leading to apoptosis (Fig. 6K).

## Discussion

Here we found that SFN inhibited microtubule-mediated FA synthesis and mitophagy resulting in decreased FA, mitochondrial dysfunction, and apoptosis. Consequently, damaged mitochondria had difficulty in maintaining their stability and protein homeostasis. These results will help us understand the underlying mechanisms that SFN triggered apoptosis in the subcellular level. HPLC-MS/MS analysis showed that FASN, ACACA, α-tubulin, and β-tubulin were the target molecules, which SFN initiated anti-cancer signaling to downregulate. Previous studies showed that SFN might bind to α-tubulin, β-tubulin to degrade α-tubulin, and β-tubulin causing apoptosis [26]. α-tubulin is the main component of microtubules and might bind tightly to β-tubulin to form microtubule dimers [27]. Microtubule acts as the cytoskeleton to support a variety of proteins for transport and scaffolding [28, 29]. Here we further uncovered that SFN inhibited the formation of dimers consisting of α-tubulin and β-tubulin, resulting in microtubule depolymerization. These results indicated that SFN-triggered microtubule disruption played a role in FA synthesis and metabolism.

Interestingly, SFN treatment caused a decrease in total free FA and mitochondrial phospholipids. Also, the levels of a few lipid enzymes involved in FA synthesis were also decreased upon SFN treatment in cultured cancer cells. FA is critical for energy metabolism and is the fundamental component of all cell membrane [30]. FASN is a key enzyme involved in the synthesis of FA essential for the de novo synthesis of FA in tumor cells [10, 31]. Therefore, it is helpful to establish a new anti-cancer therapy via targeting FA synthesis and metabolism signaling. TVB-2640 is a clinically effective FASN inhibitor, which works greatly in the treatment of patients with NSCLC [10]. However, most FASN inhibitors are not used for clinical study due to poor stability and insufficient bioavailability such as cerulenin and orlistat. It was expected that SFN would have an excellent oral bioavailability, because SFN exerted a synergistically anti-cancer effect in combination with paclitaxel reducing the dosage of both agents [32, 33]. Moreover, NSCLC patients with higher level of FASN have lower survival. By immunohistochemical staining in microarray tissues, we found that FASN was highly expressed in tumors and was associated with malignant grades of tumors. As the most critical transcription factor regulating lipid synthesis, SREBP1 is essential for the de novo synthesis of FA [34]. It was reported that ACLY, ACACA, and FASN levels were positively correlated to SREPB1 [35, 36]. Studies showed that SFN inhibited lipid synthesis by inhibiting key enzymes such as FASN and ACACA in prostate cancer [13]. Both ACACA and ACLY are the rate-limiting enzymes in de novo synthesis of FA and here their levels were decreased in response to SFN. Our results showed that SFN degraded SREBP1 and a few key enzymes in lipid synthesis through the 26S proteasome pathway. Further, knockdown of FASN caused damages in cell membrane structure and mitochondrial morphology. Mitochondria acted as the sites for ROS production and mitochondrial dysfunction induced the production of ROS. Excessive ROS might further damage the mitochondria and the accumulation of damaged mitochondria might cause apoptosis [37]. Therefore, SFN might interrupt microtubule dynamics and interfere with lipid metabolism, leading to cell death. These helped us understand SFN signaling and function in the energy metabolism.

Studies showed that the changes in membrane lipids might disrupt membrane fluidity and interfere with the normal fusion of two membrane-bound organelles, such as autophagosome and lysosome [38]. It was reported that FASN inhibitors increased the accumulation of LC3 II in a couple of cancers [39, 40]. Here, SFN inhibited FASN production, resulting in decreased levels of total free FA and mitochondrial total phospholipids and mitochondrial damage. Mitophagy served as a negative feedback maintaining cell homeostasis. As a result, SFN prevented cells from repairing damaged mitochondria by inhibiting mitophagy leading to apoptosis.

The autophagosome membrane derived from the mitochondria and the impaired mitochondrial membrane significantly prevented the formation of autophagic spots, which might be caused by a decrease in mitochondrial phospholipids. The phospholipid in the mitochondrial membrane was mainly cardiolipin, located in the mitochondrial inner membrane, and it was transferred to the outer membrane when the mitochondria was damaged, triggering mitophagy [41, 42]. Our results showed that the total free FA and mitochondrial phospholipids were increased after combined with CCCP. This suggested that SFN decreased mitochondrial phospholipid levels, thereby disrupting the mitochondrial membrane. Destruction of integrity of the mitochondrial membrane might be one of the reasons to inhibit the formation of autophagosomes and mitophagy [43]. On the contrary, decreased FA levels might inhibit mitophagy leading to apoptosis.

In NSCLC, we reported that SFN metabolites inhibited microtubule-mediated autophagy leading to apoptosis [44]. Here we found more profound mechanisms in the subcellular level. SFN mainly inhibited microtubule-mediated FA production and microtubule-regulated mitophagy affecting energy metabolism. Mitochondria acted as dynamic organelles that constantly modulated their morphology, function, and quantity to accommodate the metabolic needs of cells. Mitophagy might effectively remove damaged mitochondria by PINK/Parkin, BNIP3/NIX, and FOUNDC1 pathways [45]. The accumulation of dysfunctional mitochondria was associated with many human diseases including cancer [46]. It was reported that BNIP3L/NIX regulated the removal of the mitochondria. This mitophagy receptor bound to the mitochondria and phagocytic membrane protein LC3 II/I, and promoted mitochondrial encapsulation in autophagosomes, then it was delivered to lysosomes for degradation [46]. Both NIX and BNIP3 localized to the outer mitochondrial membrane by way of a C-terminal transmembrane domain [47]. Also, both of them directly interacted with the autophagosome membrane protein LC3 [48]. Here, we found that SFN inhibited mitophagy via downregulating BNIP3 and NIX. Further, our study showed that SFN inhibited the interaction between NIX and LC3. These results demonstrated that SFN inhibited the formation of mitophagosomes. The interaction between α-tubulin and NIX was determined after treated with SFN. After knockdown of α-tubulin, the levels of BNIP3 and NIX were downregulated, indicating that SFN induced microtubule disruption resulting in inhibition of mitophagy.

Although autophagy was discovered for more than 50 years, the relationship between mitophagy and cell death remained elusive [49]. It was reported that autophagy protected cells by maintaining energy homeostasis and nutrient requirements under starvation conditions. In tumor cells, autophagy might contribute to the undesired survival of tumor cells [50, 51]. By cotreatment with mitophagy inducer CCCP, we demonstrated that SFN decreased FA levels inhibiting mitophagy, leading to apoptosis in NSCLC cells. These gave us some new hints that SFN might disturb microtubule-mediated synthesis and mitophagy. Meanwhile, SFN might interfere with the cross-talks between FA metabolism and mitophagy.

Taken together, we uncovered a brand new mechanism that SFN induced apoptosis via inhibiting microtubule-mediated FA synthesis and mitophagy. These results might help us to establish new anti-cancer therapies via targeting lipid signaling and mitophagy-associated molecules.

## Materials and methods

### Antibodies and reagents

Anti-α-tubulin (YM3035) and anti-β-tubulin (YM3139) were purchased from ImmunoWay Biotechnology (TX, USA); anti-FASN (SC-48357), anti-NIX (SC-166332), and anti-SREBP1 (SC-13551) were purchased from Santa Cruz Biotech (TX, USA); anti-BNIP3 (D121876) and anti-ACLY (D221957) were purchased from Sangon Biotech(Shanghai, China); anti-ACACA (21923-1-AP) and anti-β-actin (66009-1-Ig) were purchased from Proteintech Group (IL, USA); anti-LC3 II/I (D3U4C) and anti-caspase-3 (D3R6Y) were purchased from Cell Signaling Technology (MA, USA); anti-VDAC1 (AB14734) was purchased from Abcam (MA, USA); D, L-Sulforaphane (574215) was purchased from Sigma-Aldrich (MO, USA); CCCP (C2759) was purchased from Sigma-Aldrich (MO, USA); Baf-A1 (S1413) and PS-341 (S1013) were purchased from Selleck (Shanghai, China); Cell Mitochondria Isolation Kit (C3601), ROS Assay Kit (S0033), and Mitochondrial Membrane Potential Assay Kit (C2006) were purchased from Beyotime Biotechnology (Shanghai, China).

### Cell line

Human lung adenocarcinoma cell line A549 and human lung squamous carcinoma cell line SK-1 were purchased and justified from the Cell Resource Center, Peking Union Medical College (Beijing, China).

### Cell culture and transfection

Cells were incubated in Dulbecco’s modified Eagle medium/F-12 culture medium with 10% fetal bovine serum (Gibco, 16,000–044) and 100 U/ml penicillin and streptomycin. All cells were cultured in a humidified incubator containing 5% CO_2_ at 37 °C.

For knockdown of FASN mRNA, negative control siRNA (5′- UUCUCCGAACGUGUCACGUTT-3′), FASN siRNA (5′-CAGAGUCGGAGAACUUGCAGGAGUU-3′), α-tubulin siRNA (5′-AAAGATGTCAATGCTGCCATT-3′), and β-tubulin siRNA (5′-CCCAGCGGCAACTACGTGGG-3′) were designed [1, 16, 24], SREBP1 siRNA was purchased from Santa Cruz Biotech (sc-36558). Cells were plated in six-well plates at a density of 1 × 10^6^/well and cultured for 24 h. Then the cells were transfected with the FASN and α-tubulin siRNA, respectively (30 pmol/well) by Lipofectamine^TM^ RNAiMAX (Invitrogen, 13778075) when cells reach ~80% confluency.

### Mitochondrial protein extraction

Cells (2 × 10^7^) treated with or without 20 μM SFN for 24 h were collected and washed once in ice-cold phosphate-buffered saline (PBS). Mitochondrial proteins were isolated from the cell by the Cell Mitochondria Isolation Kit according to the manufacturer’s instructions. Mitochondrial protein concentrations were determined by the BCA Protein Assay Kit (Invitrogen, 23227) and cytoplasm protein concentrations were determined by the Bradford assay (Applygen, P1510).

### Western blotting

Cells were lysed by RIPA buffer (Thermo Fisher Scientific, 89900) supplemented with protease inhibitors cocktail. Whole-cell lysates were quantified by the BCA Protein Assay Kit. Equal quantity of protein molecules (30 μg) from each sample were separated by 8% or 12% SDS-polyacrylamide gel electrophoresis. The procedures were carried out according to the previous experimental method [2].

### Coimmunoprecipitation

After washed with ice-cold PBS, the cells were lysed on ice via Nondenaturing Lysis Buffer supplemented with protease inhibitors cocktail. The monoclonal anti-α-tubulin or anti-β-tubulin, or anti-FASN or anti-NIX, or anti-LC3 II/I was added into the protein lysates and the mixture was incubated overnight at 4 °C. The complexes were pulled down with protein A/G agarose for 3 h and the proteins were isolated by centrifuging and boiling for 5 min. Western blotting was used to recognize the conjugated proteins.

### Immunofluorescence staining

Cells were seeded in 35 mm cover glass-bottom dishes at a density of 1 × 10^5^ cells/dish and incubated for 24 h, then treated with 20 µM SFN for 24 h. These cells were fixed with 1% paraformaldehyde for 15 min. Then the cells were washed for three times with PBST (PBS plus Tween-20) and permeabilized with ice-cold methanol for 10 min at room temperature. After blocking with PBS containing 1% bovine serum albumin and 0.1% Triton X-100 for 1 h, the cells were incubated overnight at 4 °C with the corresponding primary antibodies. Then the cells were washed for three times with PBST and incubated with the fluorescence-labeled secondary antibody for 1 h at room temperature. After washing with PBST for three times, the cells were stained with 4′,6-diamidino-2-phenylindole (Zsgb-bio, ZLI-9557). Fluorescence images were collected under a laser scanning confocal microscope (Olympus FV1000; Olympus Corp., Tokyo, Japan).

### Microtubule polymerization assay

The collected cells were washed twice with PBS, then lysed at 37 °C for 30 min with 400 μL lysis buffer (20 mM Tris-HCl pH 6.8, 1 mM MgCl_2_, 2 mM EGTA, 1% NP-40) with Protease Inhibitor Cocktail (Roche, 04693132001). The cell lysates were centrifuged at 12,500 r.p.m. for 15 min at 25 °C. The supernatant containing soluble α-tubulin was collected, whereas the pellet containing assembled α-tubulin was suspended in 40 μL of pellet lysis buffer (20 mM Tris-HCl pH 6.8, 1 mM MgCl_2_, 2 mM EGTA, 2% SDS) after washing with PBS. Then the precipitate was heated at 95 °C for 30 min until the pellet was solved. These α-tubulin molecules in two fractions (soluble and insoluble) were separated by western blotting.

### Transmission electron microscopy

Cells were treated with 20 µM SFN for 24 h. After being collected and washed with PBS for two times, the sample was fixed with 3% glutaraldehyde at 4 °C for 2 h. After washing with PBS for three times, the sample was fixed in 1% osmium tetroxide for 1 h. Samples were dehydrated through a series of concentrations of ethanol, and infiltrated and embedded in a 1 : 1 mixture of acetone and Epon-812 resin for 30 min. The samples were infiltrated in Epon-812 for 2 h, cut into ultrathin sections with a knife, and positioned on 200-mesh copper grids. Sections were stained with Uranium acetate for 30 min and then stained with Lead nitrate for 20 min. The sections were then observed and photographed with a TEM (JEM-2100Plus, JEOL, Ltd, Tokyo, Japan).

### HPLC-MS/MS analysis

HPLC-MS/MS was used to analyze the production in A549 cells after treated with or without SFN. Cells were treated with SFN for 24 h, then the cell lysates were collected and quantified. Equal quantity of protein molecules were analyzed by HPLC-MS/MS. Chromatographic separation and analysis were performed by a Orbitrap Fusion Lumos mass spectrometer (Thermo Fisher Scientific, USA) and an EASY-nLC 1000 liquid chromatography system (Thermo Fisher Scientific, USA) equipped with an electrospray ionization source. Separation was carried out by a C18 (1.9 µm, 100 Å) capillary column with a mobile phase of 0.1% HCOOH/H_2_O and 0.1% HCOOH/ACN. Each sample was injected twice to perform detection in positive ionization modes. After the collected data were identified, proteins were analyzed by the Uniprot net for cell localization and function.

### Tissue microarray immunohistochemistry

Human lung cancer tissue microarrays with 150 patient samples and different Gleason patterns were established by Shanghai Biochip (Shanghai, China). The immunohistochemistry stain was done with human-specific anti-FASN combined with the UltraSensitive™ S-P detection kit (Maixin, KIT-9710). The protocol was derived from the published paper [16].

### Apoptosis assay

Cell apoptosis was measured via Annexin V-FITC apoptosis assay kit (GenStar, C203-01). Cells were collected and washed twice with ice-cold PBS, then these cells were resuspended in 250 μL 4× binding buffer. Then 5 μL Annexin V-FITC and 10 μL Propidium Iodide were added to 100 μL cell suspension, and the reaction was incubated for 5 min at room temperature in the dark. Apoptotic cells were detected via a flow cytometer.

### Free FA quantification assay

Levels of total free FAs were determined by Free Fatty Acid Quantitation kit (Sigma, MAK044). Cells(1 × 10^6^) were collected and resuspended in 200 μL of 1% (w/v) Triton X-100 in chloroform solution. The homogenates were centrifuged at 13,000 r.p.m. for 10 min and we collected the organic phases to accept vacuum dry for 30 min, to remove trace chloroform. The dried lipids were dissolved in 200 L of Fatty Acid Assay Buffer for detection.

### Mitochondrial phospholipids quantification assay

Cells (1 × 10^6^) were collected to Isolate mitochondria with the kit. These mitochondria were lysed with 1% NP-40 for 30 min. Cell mitochondrial phospholipids were measured via Phospholipid Assay Kit (Sigma, MAK122).

### Bioinformatics analysis

GEPIA (Gene Expression Profiling Interactive Analysis) is a public database newly developed by the Chinese for cancer and normal gene expression profiling, from 9736 tumors and 8587 normal samples from The Cancer Genome Atlas and Genotype-Tissue Expression projects. We searched the GEPIA Database to determine the genetic correlation in lung cancer. The version 10.5 of STRING database was used to find the interaction among NIX and lipid metabolism-related proteins [52].

### Statistical analysis

All data were expressed as mean ± SEM from three independent experiments. Paired data were evaluated by Student’s *t*-test. Two-way analysis of variance was used to determine statistical significance. *P* ≤ 0.05 was considered statistically significant. All statistical analyses were performed by SPSS version 19.0.

## Data Availability

All data generated or analyzed during this study are included in this published article.
